# 

**Published:** 1890-10

**Authors:** 


CONTENTS—OCTOBER, 1890.—VOL. VI., NO. 4.

ORIGINAL ARTICLES— PAGE.

A New Medical College Proposed. Is It Wise? T. A. Pope, M. D .......... 139

CORRESPONDENCE—

Dr. Beall's Trip to Europe .......... 143

Letter from Birmingham. Dr. Leake .......... 158

SOCIETY NOTES—

Circular Letter to the Profession of Texas—by Committee on Legislation .......... 166

Form of Petition to Legislature .......... 174

West Texas Medical Society .......... 174

Resolution by Gainesville Medical Society .......... 175

Southern Surgical and Gynecological Association .......... 176

Cherokee County Medical Society .......... 176

EDITORIAL—

A Pull All Together .......... 177

Spots on the Sun .......... 178

The Coming Operation for Cataract .......... 180

The Transactions for 1890 .......... 181

MEDICAL NEWS AND MISCELLANY—

Numerous Items of Interest .......... 182, 176, 142

PUBLISHER'S NOTES .......... 136

Containing Valuable Information .......... 186

